# The Tumor Microenvironment of Primitive and Metastatic Breast Cancer: Implications for Novel Therapeutic Strategies

**DOI:** 10.3390/ijms21218102

**Published:** 2020-10-30

**Authors:** Giovanni Zarrilli, Gianluca Businello, Maria Vittoria Dieci, Silvia Paccagnella, Valentina Carraro, Rocco Cappellesso, Federica Miglietta, Gaia Griguolo, Valentina Guarneri, Marcello Lo Mele, Matteo Fassan

**Affiliations:** 1 Department of Medicine (DIMED), Surgical Pathology & Cytopathology Unit, University of Padua, 35121 Padua, Italy; giovanni.zarrilli@gmail.com (G.Z.); glc.businello@gmail.com (G.B.); silviapak80@gmail.com (S.P.); valentina.carraro.med@gmail.com (V.C.); rocco.cappellesso@gmail.com (R.C.); 2 Medical Oncology 2, Istituto Oncologico Veneto IOV-IRCCS, 35128 Padua, Italy; mariavittoria.dieci@unipd.it (M.V.D.); gaia.griguolo@iov.veneto.it (G.G.); valentina.guarneri@unipd.it (V.G.); 3 Department of Surgery, Oncology and Gastroenterology (DISCoG), University of Padua, 35121 Padua, Italy; federica.miglietta@iov.veneto.it; 4 Surgical Pathology Unit, University Hospital of Padua, 35121 Padua, Italy; marcello.lomele@aopd.veneto.it

**Keywords:** tumor microenvironment, stroma, tumor infiltrating lymphocytes, breast cancer

## Abstract

Breast cancer evolves thanks to a dense and close interaction with the surrounding tumor microenvironment (TME). Fibroblasts, leukocytes, blood and lymphatic endothelial cells and extracellular matrix are the constituents of this entity, and they synergistically play a pivotal role in all of the stages of breast cancer development, from its onset to its metastatic spread. Moreover, it has been widely demonstrated that variations to the TME can correspond to prognosis variations. Breast cancer not only modulates the transformation of the environment within the mammary gland, but the same process is observed in metastases as well. In this minireview, we describe the features of TME within the primitive breast cancer, throughout its evolution and spread into the main metastatic sites.

## 1. Introduction

Breast cancer (BC) is the most common malignancy in women worldwide and is responsible for 6.6% of cancer related deaths [1]. It is widely accepted that BC initiation, development and spread are conditioned by the ensemble of the surrounding stroma which, as a whole, is a complex system of cells and macromolecules accounting for the non-neoplastic component of the tumor mass (i.e., the tumor microenvironment [TME]) [2].

The TME mainly consists of fibroblasts, endothelial cells, leukocytes and extracellular matrix (ECM). All of these elements contribute a non-negligible percentage of the tumor mass and provide it with mechanical support [3]. On the other hand, they also play a role in the development of the neoplasm itself through an input/output communication mechanism with the cancer cells [3]. In particular: (i) endothelial cells are stimulated by the neoplastic cells to produce new vascular branches in order to provide oxygen and nutrients to the growing neoplastic cells; (ii) fibroblasts are activated from the neoplasm into cancer-associated fibroblasts (CAFs), so that they can produce chemokines to promote cancer cell migration and, eventually, metastasis; (iii) the extracellular matrix, through quantitative and qualitative changes, provides chemical and biomechanical signals that stimulate the survival, growth and spread of cancer cells; and (iv) macrophages and other leukocytes are also recruited for the production of soluble factors that stimulate growth, angiogenesis, migration and immunosuppression [3].

Another important concept regarding metastasis-related TME is the “seed and soil” hypothesis, initially proposed by Stephen Paget in 1889 [4]. Paget theorized that the spreading of cancer cells is neither random nor merely due to the vascular system’s anatomy: it is instead conditioned by the characteristics of each metastatic site (the “soil”) and of the cancer itself (whose circulating cells are the “seeds”). More recent studies have shown that specific cancers have the tendency to consistently metastasize to specific sites because of their organotropism. Even more surprising is the ability of the cancer to prepare a favorable environment in a distant organ before the metastasis actually takes place; this distant microenvironment is commonly called the “niche” [5].

**Figure 1:**
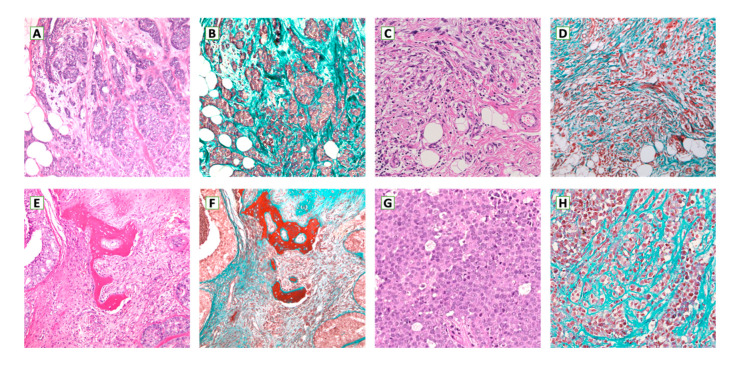
Tumor stroma in primary (**A**–**D**) and metastatic breast cancer ((**E**,**F**): bone; (**G**,**H**): brain) (**A**,**C**,**E**,**G**: H & E 100× magnification; **B**,**D**,**F**: Masson’s Tricrome 100× magnification; **H**: Masson’s Tricrome 200× magnification).

In recent years, TME has been widely linked to prognosis, and nodal and distant metastasis in various cancers (BC being one of them) and thus, it has been proposed as a potential target for cancer therapy [6]. Thus, this review focuses on the main histopathological and molecular features characterizing the TME of primitive BC, throughout its evolution and spreading into the main metastatic sites (Figure 1). We describe every component of the BC microenvironment, their evolution during BC progression, their predictive and prognostic value and the capability of the microenvironment to reproduce itself into the metastatic sites.

## 2. Primary Breast Cancer Microenvironment

The healthy mammary gland parenchyma consists of ducts and lobules, which are covered by a double-layered epithelium. The inner layer is formed by luminal cells (which have the task to produce milk) and is covered by a second layer of myoepithelial elements, whose contraction helps the emission of the milk [7]. Myoepithelial cells, in turn, lay on a basement membrane made of laminin, type IV collagen and proteoglycans and this membrane physically sets the boundaries with the surrounding stroma, which is predominantly made of type I collagen [7,8].

In BC, the composition and the architectural organization of the mammary gland parenchyma is subverted. In this context, the TME acquires a central role in cancer onset and spreading. TME in BC has been richly studied and it is now recognized as an important element that deeply affects cancer biological behavior, response to treatment, and patients’ clinical outcome [2]. The main components of the BC microenvironment are summarized in Table 1.

### 2.1. Breast Cancer-Associated Fibroblasts

CAFs are the most numerous cell population in BC stroma, and they probably represent the most important component of TME [2]. Their origin is still somewhat debated: it is likely that the majority of them derive from resident fibroblasts, reconditioned to behave in favor of the neoplastic cells. Nevertheless, some authors have suggested the hypothesis that CAFs can be also recruited from distant sites, such as bone marrow [9].

The main function of CAFs is to secrete soluble molecules to stimulate cancer cells through a paracrine mechanism: through them, CAFs interact with the neoplasm in each step of cancer development, from initiation to spreading. Here, we describe some of the molecules secreted by CAFs and the pathways they are involved in. It is interesting to note that each of these pathways are somewhat involved in just a few key mechanisms, namely epithelial to mesenchymal transition (EMT), cellular mobility and rate of proliferation.

#### 2.1.1. TGF-β1 (Transforming Growth Factor β1)

It has been shown that BC CAFs secrete TGF-β1, more than other subtypes (i.e., TGF-β2 and 3). TGF-β1 has the primary function of favoring EMT within the neoplasm, a mechanism through which cancer cells transform their phenotype into a mesenchymal-stem cell one, altering cell-to-cell adhesion, cellular polarity and extracellular matrix attachment. This process results in a more aggressive cancer phenotype, enhancing cancer cell mobility and their ability to spread [10,11].

#### 2.1.2. HGF (Hepatocyte Growth Factor)

HGF works with a paracrine mechanism. Its function requires interaction with its receptor, named c-Met. HGF affects BC development in different ways. HGF stimulates the degradation of type IV collagen via the uPA/uPAR pathway, allowing the neoplastic cells to move through the basement membrane and the extracellular matrix [12]. Furthermore, HGF promotes the up-regulation of glucose intake in cancer cells, inducing the expression of the membrane glucose transporter GLUT-1; the enhanced intake of glucose allows cancer cells to quicken their metabolism and their replication rate [13].

#### 2.1.3. IL32 (Interleukin 32)

IL32 is among the most important inflammatory mediators in the BC microenvironment [14]. It is secreted by CAFs (but not by normal fibroblasts) into the breast TME and it interacts with integrin β3, which is in turn expressed as a membrane receptor by BC cells. In cancer cells, the interaction between these two molecules causes the activation of the p38/MAPK pathway, resulting in the increased expression of molecules involved in EMT, such as vimentin, fibronectin and N-cadherin [14]. As previously mentioned, EMT enhances the ability of BC cells to migrate and spread through the extracellular matrix [14]. It has also been shown that IL32 wields a role in proliferation rate and survival of BC cells; when exposed to IL32, BC cells demonstrate a lowered rate of apoptosis together with a higher proliferation rate [15].

#### 2.1.4. IL6 (Interleukin 6)

IL6 is another important CAF-derived factor in the BC microenvironment, and works by binding to IL-6R on neoplastic cell membranes [16]. This interaction causes the activation of the JAK/STAT3 signaling pathway, which induces the homodimerization of two STAT3 molecules; the dimer enters the cells’ nucleus and works as a transcription factor [17,18]. In BC, a correlation between STAT3 activation and the transcription of genes promoting angiogenesis (i.e., VEGF and HIFα), proliferation (i.e., cyclin D1, c-Myc), EMT (i.e., vimentin, TWIST) and mobility (i.e., metalloprotease) has been found [16,17]. Moreover, the JAK/STAT3 pathway is responsible for the downregulation of HIC1 (hypermethylated in cancer 1), a tumor suppressor gene that works as a transcriptional suppressor. The downregulation of this protein contributes to further spur the cancer proliferation rate [18].

### 2.2. Breast Cancer-Associated Leukocytes

**Figure 2:**
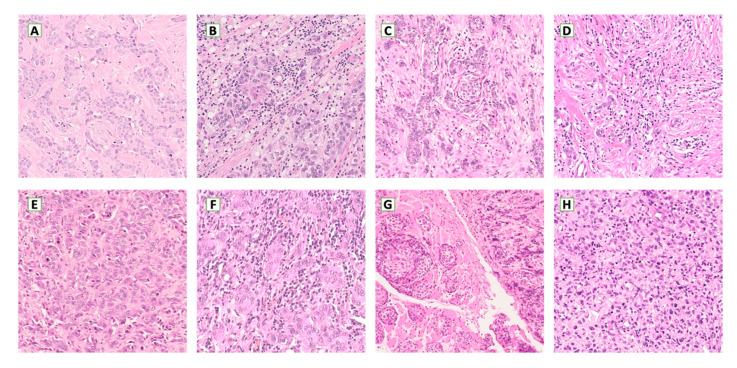
Tumor inflammation in primary (**A**–**D**) and metastatic breast cancer ((**E**,**F**): liver; (**G**): striated muscle; (**H**): brain). Low inflammatory infiltrate (**A**,**C**,**E**,**G**), high inflammatory infiltrate (**B**,**D**,**F**,**H**). (**A**,**C**,**D**,**E**,**H** H & E 100× magnification; **B**,**F**,**G** H & E 200× magnification).

Immunity has been widely recognized as a pivotal element in the development of cancer [19]. Immune cells are initially a defensive mechanism, fighting neoplastic cells. Nevertheless, cancer gains the ability to elude the leukocyte’s aggression and to activate some elements of the immune system for its benefit [7,19]. The most important leukocyte populations are represented by macrophages and lymphocytes (Figure 2) [7,19].

Macrophages are among the most abundant and important cell types within breast TME. Tumor associated macrophages (TAMs) have been shown to harbor a prognostic significance in BC; their density correlates with numerous parameters such as vascular invasion, size of the neoplasm and histologic grade; all of which cooperate to make the cancer more aggressive [7,20].

Macrophages send and receive signals from BC cells. One of the examples of this reciprocal communication lies in the paracrine CSF-1 (colony stimulating factor-1) pathway. CSF-1 is secreted by BC cells and it works as a chemotactic factor for macrophages. Macrophages expressing CSF-1 receptor are thus recruited and activated into TAMs, starting to secrete both VEGF (vascular endothelial growth factor) and EGF (epidermal growth factor) [21]. VEGF and EGF are respectively able to stimulate angiogenesis and to give metastatic potential to BC cells [21].

Another example of reciprocal communication between cancer cells and TAMs is given by the pathway that involves GM-CSF (granulocyte-macrophage colony-stimulating factor) and CCL18 (C-C motif chemokine ligand 18). This is a typical case of “loop” communication. BC cells that undergo EMT activate macrophages into TMAs, through GM-CSF; these TAMs are able to secrete CCL18, which induces EMT in BC cells, establishing a positive feedback [22].

TAMs are also known to interact directly and indirectly with other immune cells involved in the composition of breast TME. One example of this interaction involves TAM-derived IL10 (interleukin 10). This molecule directly affects dendritic cells, causing them to decrease the production and secretion of IL12, which normally works as an activator for CD8+ T lymphocytes [23]. Clearly, when IL12 is absent or decreased, there is a lack of CD8+ cell activation [23]. This pathway is important because CD8+ lymphocytes are well known to be important indicators of chemotherapy response and their absence is linked to a reduced response to therapy [23].

Another interesting mechanism through which TAMs cooperate to increase cancer malignant potential involves the protein DAB2 (disabled homolog 2). DAB2 expression seems to depend on YAP/TAZ, whose expression is in turn dependent on the extracellular matrix stiffness (*vide infra*). DAB2+ TAMs are involved in integrin turn-over. It has been shown that the presence of DAB2-negative macrophages within breast TME leads to an accumulation of integrins such as α5, α6 and β1. These integrins (through the formation of the heterodimers α5β1 and α6 β1) are involved in the interaction between neoplastic cells and some constituents of the extracellular matrix, namely fibronectin and laminin [24]. Integrins are pivotal for the tridimensional movement of cells, so that an impairment of their turn-over results in decreased cellular movement. It has been shown that BC cell lines have an enhanced invasive potential when associated with DAB2+ TAMs, even though DAB2 is not associated with the growth of the primitive tumor [24]. Furthermore, DAB2+ TAMs have been found to have a prominent role in lung metastasis. Animal models have shown that mice that do not express DAB2 have a significantly lower rate, size and number of lung metastasis [24].

Lymphocytes, and tumor infiltrating lymphocytes (TILs) in particular, represent another important yet controversial component of tumor-associated leukocytes [25]. The actual role of TILs, as a single entity, remains uncertain [26,27]. This is likely due to the intrinsic distinct biological characteristics between each subset of lymphocytes, which implies that the mere quantity of these cells is only a part of the information [28,29]. However, the simple morphological quantitative evaluation of TILs (according to the International TILs Working Group Recommendations) [30] has consistently demonstrated a strong and independent prognostic role in BC, especially for the triple-negative and HER2-positive subtypes [31,32,33,34,35,36] and their evaluation is now endorsed by international guidelines [37].

TILs are mainly constituted by T cells, and they are especially numerous in the borders of the neoplasm rather than in its core. Tumor infiltrating T cells can be further classified according to their receptor phenotype; in the BC microenvironment, CD8+ cytotoxic, CD4+ helper and regulatory T cells are the most common [38]. The value of each of these elements is still somewhat debated and their presence seems to have conflicting interpretations from one study to another [29,38,39].

CD8+ lymphocytes are known to have a tumor-suppressor role within numerous cancers. As mentioned earlier, CD8+ lymphocytes have an important predictive role in regards to chemotherapy response [30] and a prognostic one regarding overall survival (OS) [40] and disease-free survival [41].

Just like the other microenvironment cell types we have described so far, TILs need to be reconditioned by the neoplasm to behave in favor of the cancer itself (or, at least, to not behave against it) [42,43].

One way through, which seems to happen, is a TIL/TAM interaction. TAMs, as already explained before, have a crucial role in the development of BC and in the manipulation of the other TME elements. CD8+ lymphocytes are no exception, since their tumor suppressor activity is dampened by macrophages within TME with a HIF-1α (hypoxia-inducible factor-1α)-dependent mechanism [42]. The exact way this phenomenon happens is still not entirely known, but HIF-1α-depleted models show decreased tumor growth [42,43].

TILs are not only influenced by TAMs within breast TME. In cancer, as in every chronic inflammation context, the cytotoxic effect of CD8+ cells need to be modulated and muted when not necessary, to avoid damage to the surrounding healthy tissue. T regulatory cells (Treg) and FOXP3+ (forkhead box P3) guarantee the functioning of this checkpoint [44]. The balance between these elements and cytotoxic cells is pivotal to prevent the inflammatory response from proceeding unchecked and it is in fact altered in many immune-mediated pathologies [42,43,44]. Within BC, Miyashita and colleagues [44] report that neoplasms with a high number of Treg cells have a significantly poorer prognosis when compared with the ones that were scarce in FOXP3+. Furthermore, in their study, the CD8+/FOXP3 ratio was shown to be linked to recurrence-free survival (RFS) and BC specific survival: a high CD8+/FOXP3+ ratio entails a higher RFS and BCS [44]. However, the role of FOXP3+ T lymphocytes in BC is still debated. Many other studies report a positive correlation between FOXP3+ lymphocyte infiltration and improved prognosis in TNBC and estrogen receptor negative BC [45,46,47,48]. Moreover, Schmidt and colleagues reported no correlation between FOXP3 expression and BC outcome [49].

Another considerable immune checkpoint involving T cells is the PD1/PD-L1 pathway. This receptor/ligand interaction is pivotal in the modulation of T cell activity; the activation of PD-1, expressed as a receptor on T cell membranes, causes the downregulation of these cells, resulting in antigenic tolerance [50]. Neoplasms express PD-L1 to downregulate the cytotoxic effects of T cells, in order to escape the immune reaction. This behavior has been described for a series of cancers, such as melanoma, non-small-cell lung cancer, gastric cancer, colorectal cancer, cholangiocarcinoma, hepatocellular carcinoma and head and neck squamous cell carcinoma [51]. In BC, PD-L1 is predominantly expressed in immune cells (in particular in lymphocytes and macrophages) more than in neoplastic cells [52]. The incidence of PD-L1-positive expression in breast cancer has been reported as 20 to 40%, varying according to the method and the cut-off used for the assessment [53]. For triple-negative breast cancer, PD-L1 positive expression has been reported in 41% of patients with metastatic disease enrolled in the Impassion 130 randomized trial (by using the SP142 Ventana assay, with a cut-off of ≥1% of total tumor area occupied by PD-L1+ immune cells) [54]. The prognostic value of PD-L1 within BC remains somewhat unclear [50,55], and it seems to be positively linked to some clinical features of BC, such as nodal metastasis, histological grade and estrogen receptor presence [56]. Conversely, PD-L1 expression has also been associated with better RFS and OS and it may have a positive prognostic role in TNBC and HER2+ BC [53].

### 2.3. Breast Cancer-Associated Endothelium

Endothelial cells (ECs) also undergo relevant changes within the TME. First, endothelial cells need to be stimulated to produce wider vascular networks in order to provide cancer cells with oxygen and nutrients to sustain their growth. The main mechanism through which this happens is the activation of the VEGF-A pathway [57].

VEGF-A (vascular endothelial growth factor-A) is a key regulator of angiogenesis and is widely up-regulated in numerous cancers, BC being one of them [57]. VEGF-A has its main effect on ECs from veins, arteries and lymphatic vessels, promoting growth and replication, and inhibiting apoptosis [57]. In BC, it has been shown that high VEGF-A concentrations are linked to enhanced microvessel density (hemic and lymphatic), higher pathological stage and larger tumor size [57]. Furthermore, BCs with low VEGF-A concentrations are associated with higher disease-free survival and OS [57].

ECs are not only recruited from cancer cells to enhance blood flow, but they also play a role through paracrine signaling. One of these paracrine signals goes through Jag1/notch [58]. This pathway has a role in normal mammary gland development, but it also has been shown to be upregulated within various cancers, in which it enhances the neoplasm’s malignant phenotype [58]. Within breast TME, ECs are responsible for the activation of the Jag1/notch pathway. In particular, ECs express Jag1, which is a ligand for notch that is, in turn, expressed by cancer cells. The direct contact between these two populations of cells, and between Jag1 and notch, has been linked with enhanced cell proliferation, and with a more substantial metastatic potential [58].

In addition, ECs have an initial tumor suppressive role. At least one way by which ECs counteract the growth of the neoplasm is mediated by TSP-1 (thrombospondin-1) [59]. This protein is normally expressed by healthy ECs and works as a tumor suppressor [59]. Nevertheless, when the cancer-driven angiogenesis begins, the newborn ECs express a lower concentration of TSP-1 so that its tumor suppressor function is lost [59]. Moreover, in the proximity of the newborn vessels, there is an increased concentration of factors like TGF-β1 and POTSN (periostin), which are known to have a pro-tumor effect in BC [27,59].

### 2.4. Breast Cancer-Associated Mesenchymal Stem Cells

Mesenchymal stem cells (MSCs) are a population of stromal progenitors that play many pivotal roles in the maintenance of a tissue’s homeostasis. In fact, they favor the repair and neovascularization processes after tissue damage; they guarantee immune modulation, and harbor the ability of self-renewal and to generate different types of specialized stromal cells, such as fibroblasts and fibro-vascular stromal cells [60,61].

Within BC, they are actively recruited from bone marrow and adipose tissue [60,62]. An interesting way through which MSCs interact with BC is via exosomes. Exosomes are membrane-covered vesicles that contain molecules such as proteins, enzymes and nucleic acids; these vesicles are used by cells to send chemical signals [63]. MSCs have been found to generate exosomes within the BC microenvironment; through this mechanism, BC cells were stimulated to proliferate, to migrate and they were less susceptible to drug-induced apoptosis when compared to controls [64]. Another way through which MSCs promote a pro-tumor environment is by conditioning immune cells. In particular, MSC-derived exosomes have been found to contain molecules (such TGF-β), which are able to induce the transformation of monocytic myeloid-derived suppressor cells into M2-polarized macrophages; it is interesting to note that no other cell type is known to produce exosomes with similar features [65]. M2-polarized macrophages are considered immunosuppressive cells, even though this is an oversimplified definition [66]. The polarization of macrophages towards the M2 subtype was found to be linked to a de-repression of PD-1 expression in TILs [65]; as we mentioned in Section 2.2, PD-1′s activation in TILs is well known to cause downregulation of these cells, with resulting antigenic tolerance [50]. To summarize, this exosome-mediated pathway allows MSCs to modulate the behavior of macrophages, which in turn modulates the behavior of TILs to obtain, as a result, a down regulated immune response to BC cells with a “doubly indirect” mechanism [65].

Furthermore, MSCs have been found to interact with BC cells in an intriguing, yet still poorly understood way, namely dormancy through cell cannibalism [67].

The term dormancy is used to describe at least two distinct conditions, namely population level- and cellular-dormancy; the former is referred to as a micrometastatic focus in which tumor replication and death are in balance, with no net variation in the population itself; the latter is instead a condition in which cancer cells enter the G0 state [68]. Dormant cancer cells are thought to be responsible for cancer relapse after “awakening” from the dormant state [67]. Cannibalism, on the other hand, is one of the cell-in-cell structures (the others being entosis and emperitosis); through cannibalism, a neoplastic cell, triggered by starvation, internalizes and catabolizes another alive or dead cell [69]. BC cells have been shown to cannibalize MSCs within the tumor microenvironment; this phenomenon results in the death of the internalized MSC and in a mutation in the behavior of the neoplastic cell, which gains enhanced survival potential after starvation and, at the same time, loses part of its tumorigenic potential: in short, BC cells enter dormancy [67].

Nevertheless, Chen and colleagues [70] reported a different outcome that follows MSCs’ cannibalization by BC cells; they found that this interaction could potentiate the stemness, migration, invasion, and metastatic ability of BC. Moreover, they found that MSC cannibalization leads to significant changes in the gene expression profiles of BC with upregulation of oncogenic pathways (e.g., Wnt, p53, C-MYC, and TGF-beta) and cell membrane and matrix-associated proteins (e.g., integrins, syndecan). Despite the role of cannibalism in BC, it is not clear yet whether it represents an intriguing field for new studies.

### 2.5. Breast Cancer-Associated Extracellular Matrix

The extracellular matrix (ECM) consists of an intricate ensemble of molecules, whose organization in 3D space is essential to provide a framework to the cells that compose a tissue. Furthermore, ECM is also important to guarantee a series of chemical and biomechanical signals that allow the proper functioning of a tissue, cancer included [71].

Just like the other component of the TME, the ECM is a dynamic element, it changes according to the state of development of the cancer, which is in turn conditioned by ECM composition [71].

During cancer development, breast ECM undergoes some important modifications to the composition of its main components; there is an increase in type I, II and V collagen and glycosaminoglycans, while type IV collagen and laminin-111 (LM-111) decrease [72,73].

One of the most important families of proteins that affect the composition and the organization of breast TME is constituted by the MMPs (matrix metalloproteinases) [27]. These proteins’ main ability is to degrade the constituents of the ECM [27]. Within mammary parenchyma, we can divide the EMC into two major components: basement membrane (BM) and interstitial stroma [7].

The BM is the first obstacle for the spreading of BC, so that its overcoming defines an invasive neoplasm. BM is made mostly by type IV collagen, laminin and proteoglycans [7,73]. The most important MMPs involved in the transition through BM are MMP-2, 7 and 9, which have in common a type IV collagen degrading ability [74]. Furthermore, the high expression of at least MMP-2 and 9 has been found to be linked to a poor prognosis, together with higher levels of MMP-1, 12, 14 and 15 [7,75]. In contrast, none of the MMPs seem to have a positive prognostic impact [74,75].

Collagen degradation is a well-known way by which ECM helps cancer spreading [72,76]. Nevertheless, collagen can be positively associated with cancer mobility and spreading through morphological alterations. It has been shown, in fact, that collagen goes through three stages of development, named TACS (tumor-associated collagen signatures), from tumor initiation to its spreading within and outside mammary parenchyma [7,76]:

-TACS-1 corresponds to the first stage of collagen organization around the arising tumor and it is already present in a very precocious stage of cancer development. TACS-1 consists of an increased deposition of muddled collagen in the proximity of the neoplasm.

-TCAS-2 is seen when BC increases in size; at this point, collagen fibers are stretched, and their axis is parallel to the edges of the growing neoplasm.

-TACS-3 can be considered as collagen organization that promotes the invasive phenotype of the cancer. In this phase, collagen fibers are reoriented to be perpendicular to the neoplastic mass. This shape provides an eased path for BC cells to infiltrate through.

The deposition of collagen in the form of TACS-3 has also been associated with a poor prognosis [77].

## 3. Engineered Breast Cancer Models

One of the most relevant problems in the study of TME is represented by the construction of a representative model. Nowadays, a cancer model can be reproduced through different methods, namely: xenograft, spheroids, organoids, microfluidic technology, tissue culture plate and fibrous scaffold [78]. Each of these surrogates have some strength and specific features, but to date none of them can perfectly reproduce a TME [78].

Xenograft is a technique through which neoplastic human cells are implanted in an animal model, usually a mouse [78]. It represents an *in vivo*, three-dimensional (3D) model. Nevertheless, xenografts harbor some intrinsic issues, such as the fact that human stroma within the neoplasm is rapidly replaced by host stroma: this occurrence can cause some, even possibly slight, alterations in the cancer behavior [78]. Furthermore, xenografts are expensive, and a long period of time is needed to make a successful implant [78].

Organoids represent 3D in vitro structures and are composed of agglomerates of cancer cells supported by a matrix structure (e.g., Matrigel) [79,80]. These structures have the peculiar features of reproducing the shape of the primitive tumor, developing different cell subtypes and behaving like the primitive cancer [80]. There is also the possibility to create spheroids with a mixed cellular composition, such as cancer cells and CAFs. The main limitations in the use of this technique are the low reproducibility and the impossibility to reproduce all of the elements of the primitive stroma and their reciprocal interactions [79,80,81].

Microfluidic models consist of a 3D, *in vitro*, liquid substrate in which neoplastic cells are suspended [78]. This technique is highly automated, and allows study of EMT, the interaction between cancer cells and endothelial cells, and the movement of cancer cells in response to molecule gradients [78]. The major pitfall of microfluidic models is the impossibility to recreate the whole microenvironment, and their tendency to excessively simplify a complex biological system [78].

Tissue culture plates represent an economic, simple and relatively rapid way for growing cancer cells. However, this 2D in vitro technique has some important issues, because it is impossible to properly study stroma and intercellular interactions [79].

Fibrous scaffolds consist of 3D, in vitro models that replicate the features of the primitive cancer stroma. They are mainly made of natural, animal-derived components such as type I collagen, fibrin and Matrigel and/or synthetic biomaterials such as PLG, PLA, PLGA and PEG [82]. The main advantage in these techniques is the possibility to modulate the characteristics of each substrate according to the specific conditions of the single study; this allows one to study efficiently the interactions between different components of a TME. Unfortunately, fibrous scaffolds have the limitation of intrinsic batch-to-batch variability, which makes every experiment not completely and perfectly reproducible [78].

**Table 1:** Here we have a summary of the above-discussed components of the TME with their main pro-tumoral functions.

TME Component	Main Functions	Ref.
Fibroblasts	Promotion of EMT. Enhancement of proliferation rate. Induction of ECM remodeling. Lowering of apoptotic rate. Promotion of angiogenesis.	[10,11,12,13,14,15,16,17,18]
Macrophages	Promotion of angiogenesis. Immunosuppression. Promotion of EMT. Enhancement of cancer motility.	[21,22,23,24]
Lymphocytes	Deregulation of immune checkpoints in favor of immunosuppression.	[24,25,26,27,28,29,30,31,32,33,34,35,36,37,38,39,40,41,42,43,44,45,46,47,48,49,50,51,52,53]
Endothelial cells	Angiogenesis Enhanced proliferation rate and metastatic potential through paracrine signaling.	[57,58,59]
Mesenchymal stem cells	Enhancement of proliferation rate via exosomes. Immunosuppression. Induction of dormancy through cannibalization.	[60,61,62,63,64,65,66,67,68,69]
Extracellular matrix	Enhancement of cancer motility.	[71,72,73,74,75,76,77]

## 4. Interaction Mechanisms in the Breast Cancer Tumor Microenvironment

A complex network of interactions occurs between cancer cells and TME protein and cellular components. For example, chemokine receptor complexes are one of the major actors in extracellular signaling processes, present both on cancer and TME cells [83]. In BC, the interaction between the chemokine receptor CXCR4 with its ligand CXCL12 can activate multiple signaling pathways, such as PI3K/AKT, Src/ERK1-2, NF-kB, STATE-3 and cross talk between CXCR4 and NOTCH, Wnt and SHH networks [84]. Through these mechanisms, the CXCL12/CXCR4 axis promotes BC cell growth, progression, angiogenesis, invasion, adherence and migration [85].

The cadherin superfamily is another important player involved in cell-to-cell and cell-to-ECM constituent adhesion and is composed of protocadherins, cadherins, desmocollins, desmogleins, contactins, and connexins [86]. In BC, cadherins and in particular E-cadherin, are involved in the process of EMT and mesenchymal to epithelial transition (MET) [87].

Paracrine interaction between cancer cells and TME components is a recognized mechanism of interaction in BC and has already been reported in this text (*vide supra*). This crosstalk between neoplastic cells and TME plays a role in BC progression and has been related to poor prognosis [88].

ECM remodeling could also be referred to as a crucial process of interaction between BC cells and TME; ECM modifications are discussed above in this text (vide supra).

Among others, mechanotransduction represents one of the most important processes involved in breast carcinogenesis.

Mechanotransduction includes all the cellular processes that transform mechanical inputs in biochemical signals, permitting the cells to adapt their physical background. These processes are involved in many different physiological and pathological processes, such as embryogenesis, atherosclerosis and neoplastic diseases [89]. In particular, understanding the role of mechanotransduction and its molecular mechanism in cancer is one of the most challenging fields in biomedical research. There is increasing evidence showing that changes in ECM stiffness, ECM remodeling and the resulting interference in cytoskeletal tension and mechanotransduction signaling pathways can promote malignant transformation, tumorigenesis, angiogenesis, migration and metastasis [90,91,92,93]. Alterations in tensional forces generated by the actin-myosin cellular apparatus and, more in general, cytoskeletal reorganization, play a central role in the acquisition of a malignant phenotype by neoplastic cells [89]. The Rho family GTPases are one of the main cytoskeletal tension regulators, among many other functions: through Rho kinase (ROCK), Rho regulates myosin light chain phosphorylation and myosin phosphatase phosphorylation, generating contractile forces and modulating cytoskeletal tension [89]. Paszek and colleagues [94] studied the relationship between tissue rigidity and mammary tumor behavior at the molecular level in 3D matrices. According to their results, the exogenous forces represented by the matrix stiffness and the endogenous force produced by the cytoskeletal tension are part of a “mechano-circuit” that modulates malignant transformation in tumors, coupling the mechanosensory integrin pathway to Rho and ERK signaling pathways [89,94]. The increase in matrix stiffness and, consequently, of exogenous forces cause the aggregation and clustering of integrins, resulting in augmented Rho–ROCK-dependent cytoskeletal tension, which amplifies the formation and stabilization of cell-matrix adhesion assemblies. The augmented cellular endogenous forces induce cell-matrix adhesion kinase signaling, ROCK-mediated disruption of adherens junctions and growth-factor-dependent ERK activation, resulting in: (i) tumor cell proliferation, (ii) disruption of basal polarity, (iii) abrogation of glandular lumen formation, and (iv) remodeling of mammary tissue architecture [89,94]. On the other hand, bringing back cytoskeletal tension to normal levels through impairment of Rho/ERK signaling resulted in a significant reduction in tumor cell proliferation and repression of the malignant phenotype [94]. Both integrins and Rho-mediated regulation of intracellular tension are considered necessary to promote the invasive phenotype of fibroblasts and cancer cells in co-cultures [95,96].

YAP and TAZ are other well-known nuclear transducers of cell mechanics, able to transform different biomechanical signals into biological effects, in a manner that is specific for each type of cell and mechanical stress [97]. YAP/TAZ are involved in many different physiological and pathological processes, and a complete discussion of their mechanobiology is far beyond the purpose of this review [97]. The role of YAP/TAZ is better understood in the context of the Hippo pathway, which is deregulated in different human cancers [98]. The serine/threonine kinases MST1/2 and LATS1/2 have a central role in the Hippo pathway. Together with the adaptor proteins SAV1 and MOB1/2, MST1/2 and LATS1/2 phosphorylate the downstream effector proteins YAP and TAZ, which are thus sequestrated in the cytoplasm by 14-3-3 proteins [99,100]. Moreover, YAP/TAZ phosphorylation induces their degradation via the proteasome, which is precipitated by the F-box protein family member β-TrCP [101]. These regulatory mechanisms prevent YAP/TAZ from entering the nucleus, where they can bind to the TEAD transcription factors, inducing the transcription of genes involved in cellular proliferation and survival such as *CTGF*, *CRY61*, *BIRC5*, *ANKRD1*, and *AXL* [98]. YAP/TAZ can also cooperate with the transcription factors RUNX1/2, TBX5 and SMAD [100,102]. YAP and TAZ are commonly induced in many human cancers, BC included [103,104]. Aberrant tissue organization, accumulation of stromal cells, inflammation, increased compression forces and interstitial pressure, metalloprotease-mediated ECM remodeling by CAFs and overall ECM stiffening are considered the pivotal inputs that induce YAP and TAZ overactivation in cancer cells [97]. In BC patients, elevated YAP/TAZ activity has been associated with high histological grade, enrichment of stem cell signatures, metastasis propensity and poor clinical outcome [104]. Furthermore, the Hippo pathway represents a promising target for BC therapies [105]. The role of YAP in BC has not been clearly elucidated yet, and more studies are needed to define how this molecule influences mammary carcinogenesis. Lamar and colleagues [106] reported that, through its TEAD-interaction domain, YAP enhances the processes of cellular proliferation, migration and metastatic invasion in breast cancer cells. Overholtzer and colleagues [107] suggest that overexpression of YAP in human non-transformed mammary epithelial cells induces anchorage-independent growth, EMT, growth factor-independent proliferation, activation of AKT and ERK, pathways and inhibition of apoptosis. Notably, the study of Lee and colleagues [108] in 3D BC models establishes that BC progression is regulated by a YAP-independent mode of mechanotransduction. Moreover, Chen and colleagues [109] suggest that YAP hyperactivation alone is insufficient to drive oncogenic growth in the normal mammary epithelia and probably requires cooperating genetic alterations.

Conversely, the role of TAZ in BC progression, and its clinical implications, seems to be better understood as. Bartucci and colleagues [110] suggested that TAZ overexpression in dedifferentiated BC cells confers a malignant phenotype and migratory activity, while loss of TAZ in BC stem cells compromises metastatic colonization and chemoresistance. Moreover, they reported that high TAZ expression was associated with shorter disease-free survival in 99 BC cancer patients. The association of TAZ overexpression and poorer clinical outcomes has also been reported by Diaz-Martin and colleagues [111]. Higher levels of TAZ mRNA and/or protein expression seems to be more frequent in TNBC than in other histotypes [104,111,112,113]. Furthermore, TAZ has been proposed as a predictor of pathological complete response in Luminal B and HER2-positive breast cancer patients treated with neoadjuvant chemotherapy and Trastuzumab [114].

Finally, Yoon and colleagues [115] described the role of COX-2 expression, signaling and function in the induction of physical forces in human BC, linking the regulation of COX-2-PGE2-EP signaling with mechanotransduction and the physical properties of the tumor microenvironment.

## 5. Metastatic Breast Cancer Microenvironment

The TME not only plays a pivotal role within the primitive site of cancer growth, but it is also a leading factor for metastasis [116]. As already mentioned, there is a pre-conditioned metastatic environment within distant organs prepared to receive cancer cells. The adjustment of the distant environment is supposedly mediated by circulating fibroblasts, which are thought to be able to leave the primitive cancer site to colonize distant organs. It has been shown that patients with metastatic BC have a high incidence of tumor-derived circulating fibroblasts, which can travel through the bloodstream as single cells, as a bundle of fibroblasts or associated with cancer cells [116]. These fibroblasts seem to be responsible for the setting of a favorable microenvironment for the cancer (i.e., the metastatic “niche”) [116]. Within a niche, the newly arrived cancer cells start to shape the environment to make it suitable for its growth. Various examples of this mechanism have been described, even though many aspects of the niche-associated metastasis have to be elucidated.

Within the liver, metastatic cells seem to induce cytokine production from Kupffer cells, which in turn enhance the adhesive properties on the lumen of sinusoidal endothelium [117].

It has also been shown that BC metastatic cells within the liver niche regain E-cadherin expression. This allows the cancer cells to go back towards an epithelial phenotype through MET [118]. The signaling pathway associated with E-cadherin expression is due to the liver microenvironment, even though this exact mechanism within BC is not entirely understood [118].

Within the brain, BC cells need to shape the local stroma in order to support the neoplasm’s growth. BC brain metastases probably represent the most severe complication of this neoplasm, leading to poor prognosis and reducing the quality of life [119].

The mechanism of brain metastasis seems to involve NPCs (neural progenitor cells), which are recruited to the site in which the metastasis takes place. These cells play an initial tumor-suppressor role, but the neoplastic cells can progressively induce the differentiation from NPC to astrocyte via the BMP-2 (bone morphogenetic protein 2) pathway [120]. Astrocytes within the BC brain metastasis niche are known to be associated with an increased expression of genes such as BCL2L1 and TWIST1, which are considered pro-survival genes [120].

Bone is another typical site of metastasis for BC [121,122]. Bone metastatic BCs are usually associated with osteolytic lesions [121]. In these kinds of lesions, the bone matrix is disrupted by activated osteoclasts, which are the one cell-type able to metabolize the bone’s matrix. The activation of osteoclasts has been shown to be mediated by cancer cells via the Jag1/notch pathway. Within the bone niche, cancer cells have been found to express Jag1, which binds its receptor notch, expressed by pre-osteoclasts [121,122]. This interaction leads to the evolution from pre-osteoclasts to osteoclasts, which are now able to degrade the bone extracellular matrix, within which are embedded growth factors, such as TGF-β. TGF-β enhances the aggressive phenotype in the neoplasm, so that this pathway can go on fueling itself [121,122].

## 6. Relationship between the Microenvironment and the Molecular Subtypes of Breast Cancer

On the basis of gene expression patterns, BC can be classified into four molecular subgroups: Luminal A, Luminal B, HER-2 enriched and triple-negative [123]. TNBCs do not express estrogen receptors (ERs), progesterone receptors (PRs) or HER-2 [124]. Basal-like breast cancers (BLBCs) form a category of BCs that express basal markers, such as keratins 5,6 and 17, and show a phenotype similar to TNBC [125,126]. Despite the similarities and the fact that most TNBCs are also BLBCs and vice versa, the two entities are not biologically synonymous [127,128]. TNBCs represent 10–15% of BC and are associated with a poor prognosis. Moreover, they cannot be treated with endocrine or HER-2 targeted therapies [38]. Due to the fundamental role of TME in BC carcinogenesis and the lack of specific drugs against TNBC, many studies have tried to shed light on peculiar characteristics of the TNBC microenvironment in order to discover new clinical biomarkers or therapeutic targets [38].

TIL infiltration tends to be higher in ER negative BC and in particular in TNBC [129,130]. A prominent T cell population is common in TNBCs and BLBCs, and TNBC seems to be characterized by a higher density of CD8+ TILs [131,132]. Moreover, TNBC has the highest average number of FOXP3+ T lymphocytes compared to the other BC subtypes [29,133]. The number of FOXP3+ positive T cells seems to be associated with tumor aggressiveness, since BLBCs have the highest FOXP3+/CD8+ T lymphocytes ratio and luminal A has the lowest [133,134].

Glajcar and colleagues [135] report that luminal A and luminal B tumors have significantly higher numbers of intratumoral chymase- and tryptase-positive mast cells compared to triple-negative and HER2+ non-luminal lesions. In their study, a denser mast cell infiltration has been associated with lower tumor grade, higher ER and PR expression, lower proliferation rate and lack of HER2 overexpression.

Medrek and colleagues [136] suggest that CD163+ and CD68+ macrophages in tumoral stroma have clinical importance. In particular, CD163+ macrophages positively correlate with higher grade, larger tumor size, Ki67 positivity, estrogen receptor negativity, progesterone receptor negativity, and TNBC/BLBC subtypes, and inversely correlate with the luminal A subtype, while CD68+ macrophages correlate with tumor size and inversely correlate with the luminal A subtype [136]. Sousa and colleagues [137] report that CD163+ M2-macrophages are significantly associated with higher proliferation rate, poor differentiation, estrogen receptor negativity and the histological ductal type. Furthermore, Hollmén and colleagues [138] found that granulocyte colony stimulating factor was highly expressed in TNBC and was significantly associated with CD163+ macrophages, poorer OS and increased numbers of TGF-α+ cells.

Levano and colleagues [139] report that BLBC cell lines preferentially express molecules associated with tumor invasion and metastasis in response to macrophage-derived cytokines, such as hepatocyte growth factor receptor (HGFR), CD44, epithelial growth factor receptor (EGFR), oncostatin M receptor (OSMR) and transforming growth factor receptor 2 (TGFBR2), when compared to luminal cell lines.

Niemiec and colleagues [140] found that a significantly higher lymphatic vessel density and podoplanin expression in stromal fibroblasts are associated with (i) high grade tumors, (ii) triple-negative carcinomas, (iii) tumors expressing CK5/6, SMA, or P-cadherin, and (iv) neoplasms with stroma intensively infiltrated by lymphocytes. On the other hand, they observed a significant inverse relationship between the expression of podoplanin in the luminal A subtype, P-cadherin, CK5/6, and SMA-negative BC and in tumors without strong lymphocytic infiltration.

Intratumoral VEGF levels have been reported to be higher in TNBC compared to non-TNBC subtypes, and this seems to have a prognostic significance [141,142,143].

Finally, a different metastatic pattern has been observed in different BC subtypes. Luminal A tumors are prone to metastasize to the bone, while BLBC tends to metastasize to the brain and lung [144,145,146,147].

## 7. Therapeutic Implications

TME is a pivotal element in each of the phases of development of BC, so that its impairment can result in an efficient way to lower its aggressivity and malignant progression [2].

TILs and their tumor suppressing activity are among the most important potential targets within the microenvironment. As previously discussed, the immuno-suppressive pathway involving PD-1/PD-L1 is one of the most important ways through which cancer cells escape the immune response. Therapies that affect this checkpoint have been found to be effective in numerous cancers, such as melanoma, non-small cell lung cancer, head and neck squamous cell carcinoma and gastric cancer. In breast cancer, there have been trials with antibodies that interact with both PD-1 (Pembrolizumab and Nivolumab) and PD-L1 (Avelumab and Atezolizumab) [148]. To date, the data we have on BC and PD-1/PD-L1-based therapies are promising: activity has been shown for all the mentioned antibodies, even though the overall response rate was found to be highly variable; this is probably also due to the lack of a standardized method to select the BC cohorts that can benefit from these therapies [148]. The combination of nab-Paclitaxel and Atezolizumab as first line therapy for metastatic triple-negative breast cancer patients with PD-L1 expression ≥1% on immune cells (by SP142 Ventana assay) has been approved by both FDA (Food and Drug Administration) and EMA (European Medicines Agency) based on the positive results of the Impassion130 randomized trial [54].

The CSF-1 pathway has also been proposed as a target for immunotherapy. In animal models, it has been shown that antibodies able to inhibit CSF-1 receptors improve survival by reducing the growth of primary cancer and the rate of pulmonary metastasis better than chemotherapy alone [2,149].

Other studies have tried to target TGF-1β’s receptor in order to impair the CAF/cancer-cells axis. Animal models treated with anti TGF-1β’s receptor 2 [150] and receptors 1,2,3 [151] in combination with traditional chemotherapy showed an improved ability to reduce cancer growth and metastatic potential, and to enhance antitumor immunity.

A large body of preclinical and clinical evidence consistently suggests that the antitumor effect of conventional chemotherapy may be partly due to the modulation of the tumor immune microenvironment, resulting in the restoration of immunosurveillance. In this context, one of the most relevant mechanisms through which chemotherapy targets the immune system is represented by immunogenic cell death (ICD). ICD is a type of tumor cell death capable of priming the antitumor immune response through the exposition of calreticulin by dying tumor cells and the subsequent release of damage-associated molecular pattern molecules, thus promoting the optimal antigen presentation by DCs to T cells, and ultimately triggering the cytotoxic immune response against the remaining tumor cells. Several chemotherapeutic agents are known to be capable of inducing ICD, including, among others, anthracyclines, cyclophosphamide, platinum salts, and gemcitabine, which are routinely used for the management of BC patients [152].

Similarly, radiotherapy, which represents a cornerstone of BC locoregional management both in the curative and palliative setting, has been reported as capable of targeting the BC immune microenvironment by promoting cross-priming and by eliciting a T-cell immune response against cancer cells [153].

Interestingly, these concepts may acquire further relevance in the current era of immunotherapy, where the modulation of the immune system induced by chemotherapy or radiotherapy may be exploited as a priming strategy aiming at improving the efficacy of immunotherapy itself. Indeed, a recent adaptive non-comparative phase II clinical trial, evaluated the activity of the immune checkpoint inhibitor Nivolumab after induction therapy with either hypofractioned irradiation or various chemotherapeutic agents, even as single agents, in a cohort of patients with advanced triple-negative breast cancer. This study provided evidence that induction treatment with either doxorubicin or platinum salts is able to induce a more inflamed TME, thus enhancing response rates following immunotherapy [154]. Although promising, these preliminary results deserve further validation in the context of larger and properly designed clinical trials.

Another interesting way through which the microenvironment is targeted for therapeutic purposes, is represented by endocrine treatment, which is thought to modulate the immune system to varying degrees. Endocrine therapies, including selective estrogen receptor modulators (SERMs, e.g., Tamoxifen), selective estrogen receptor down-regulators (e.g., Fulvestrant) and aromatase inhibitors (AIs, e.g., Letrozole, Anastrozole, Exemestane) currently represent the backbone of HR+ BC management both in the early and advanced setting. Preclinical and clinical evidence suggests that SERMs and AIs may foster the anti-tumor immune response through several mechanisms. In particular, SERMS have been reported to be capable of decreasing the intratumoral levels of CCL2 and CCL5 in preclinical models, thus promoting TAM polarization towards the M1 phenotype, ultimately enhancing the anti-tumor immune system [155]. In addition, accumulating evidence from both preclinical and clinical studies suggests that AIs may modulate the immune infiltrate composition in the context of BC TME by hindering naïve T-cell differentiation into T-regulatory cells (FOXP3+ T cells), resulting in a more favorable CD8+/FOXP3+ ratio [156,157]. However, it has been suggested that endocrine therapy may also have a role in the opposite direction. Indeed, SERMs have been reported to be capable of promoting an immunosuppressive milieu in the context of BC TME [158,159,160] by (i) inducing CD4+ T-cell polarization towards a Th2 phenotype through the inhibition of DC differentiation, maturation and function, (ii) suppressing the cytotoxic immune activity through the inhibition of CD8+ T cells.

In the light of the inconsistency of available evidence, it is not possible to draw definitive conclusions on the actual role of endocrine therapy in targeting BC TME.

The bone microenvironment has also been proposed as a target for BC therapy. Bisphosphonates are the main class of drugs used to modulate the bone matrix. In BC, their effect has been shown to be most important for post-menopausal women, in which they lower mortality and both local and distant recurrence [161]. Another way to impair the bone microenvironment is through Denosumab, a monoclonal antibody that can bind and inhibit RANKL (nuclear factor-κB ligand), an essential cytokine for the maturation and proper function of osteoclasts [162]. Patients who received Denosumab in the metastatic setting showed an overall survival similar to those who received bisphosphonates and a lower rate of skeletal-related events, such as fractures and hypercalcemia [162].

Albeit conceptually intriguing, unfortunately none of the TME-based therapies have shown brilliant effects against BC so far. This is possibly due to the currently weak classification of the different features in individual patients’ TME and to the impossibility to set up a real targeted therapy.

## 8. Conclusions

BC represents the most common cancer in women and one of the main cancer-related causes of death. There is increasing evidence that the tumor microenvironment plays a pivotal role across all of the stages of development of BC.

In this review, we highlighted the main entities that constitute TME, cellular and non-cellular ones, and the fundamental ways through which these entities are involved in the initiation, progression and spread of the cancer. Of course, the pathways involved within any given TME are intertwined and their actual working is more complicated than we described. Not only is it established that TME is fundamental for a cancer to grow but is now known that it also has implications upon the prognosis and the response to therapy.

It is fascinating to see how cancer cells, in order to survive and spread, need to condition all of the surrounding tissue to “cooperate”; this evidence promotes the well-established concept that cancer is not a group of cells, it is a tissue.

Even more fascinating, and somewhat disturbing, is the evidence that cancer cells can promote the making of an ad hoc environment into metastatic sites before and after actually invading them, even though the mechanism through which this happens is far from being completely elucidated.

The study of TME not only gives us insight into the development of a neoplasm, but it is also a source of information to elaborate novel optimized and personalized cancer therapies.

## References

[1] Bray F., Me J.F., Soerjomataram I., Siegel R.L., Torre L.A., Jemal A. (2018). Global cancer statistics 2018: GLOBOCAN estimates of incidence and mortality worldwide for 36 cancers in 185 countries. CA A Cancer J. Clin..

[2] Soysal S.D., Tzankov A., Muenst S.E. (2015). Role of the Tumor Microenvironment in Breast Cancer. Pathobiology.

[3] Wang J.-J., Lei K.-F., Han F. (2018). Tumor microenvironment: Recent advances in various cancer treatments. Eur. Rev. Med. Pharmacol. Sci..

[4] Paget S. (1989). The distribution of secondary growths in cancer of the breast. Cancer Metastasis Rev..

[5] Kaplan R.N., Riba R.D., Zacharoulis S., Bramley A.H., Vincent L., Costa C., Macdonald D.D., Jin D.K., Shido K., Kerns S.A. (2005). VEGFR1-positive haematopoietic bone marrow progenitors initiate the pre-metastatic niche. Nat. Cell Biol..

[6] Roma-Rodrigues C., Mendes R., Baptista P.V., Fernandes A.R. (2019). Targeting Tumor Microenvironment for Cancer Therapy. Int. J. Mol. Sci..

[7] Conklin M.W., Keely P.J. (2012). Why the stroma matters in breast cancer: Insights into breast cancer patient outcomes through the examination of stromal biomarkers. Cell Adhes. Migr..

[8] Polyak K., Kalluri R. (2010). The Role of the Microenvironment in Mammary Gland Development and Cancer. Cold Spring Harb. Perspect. Biol..

[9] LeBleu V.S., Kalluri R. (2018). A peek into cancer-associated fibroblasts: Origins, functions and translational impact. Dis. Model. Mech..

[10] Roche J. (2018). The Epithelial-to-Mesenchymal Transition in Cancer. Cancers.

[11] Yu Y., Xiao C.-H., Tan L.-D., Wang Q.-S., Li X.-Q., Feng Y.-M. (2013). Cancer-associated fibroblasts induce epithelial–mesenchymal transition of breast cancer cells through paracrine TGF-β signalling. Br. J. Cancer.

[12] Jedeszko C., Victor B.C., Podgorski I., Sloane B.F. (2009). Fibroblast Hepatocyte Growth Factor Promotes Invasion of Human Mammary Ductal Carcinoma In situ. Cancer Res..

[13] Brauer H.A., Makowski L., Hoadley K.A., Casbas-Hernandez P., Lang L.J., Roman-Perez E., D’Arcy M., Freemerman A.J., Perou C.M., Troestere M.A. (2013). Impact of tumor microenvironment and epithelial phenotypes on metabolism in breast cancer. Clin. Cancer Res..

[14] Wen S., Hou Y., Fu L., Xi L., Yang D., Zhao M., Qin Y., Sun K., Teng Y., Liu M. (2019). Cancer-associated fibroblast (CAF)-derived IL32 promotes breast cancer cell invasion and metastasis via integrin β3–p38 MAPK signalling. Cancer Lett..

[15] Wang S., Chen F., Tang L. (2014). IL-32 promotes breast cancer cell growth and invasiveness. Oncol. Lett..

[16] Masjedi A., Hashemi V., Hojjat-Farsangi M., Ghalamfarsa G., Azizi G., Yousefi M., Jadidi-Niaragh F. (2018). The significant role of interleukin-6 and its signaling pathway in the immunopathogenesis and treatment of breast cancer. Biomed. Pharmacother..

[17] Banerjee K., Resat H. (2016). Constitutive activation of STAT3 in breast cancer cells: A review. Int. J. Cancer.

[18] Sun X., Qu Q., Lao Y., Zhang M., Yin X., Zhu H., Wang Y., Yang J., Yi J., Hao M. (2019). Tumor suppressor HIC1 is synergistically compromised by cancer-associated fibroblasts and tumor cells through the IL-6/pSTAT3 axis in breast cancer. BMC Cancer.

[19] Ravelli A., Roviello G., Cretella D., Cavazzoni A., Biondi A., Cappelletti M.R., Zanotti L., Ferrero G., Ungari M., Zanconati F. (2017). Tumor-infiltrating lymphocytes and breast cancer: Beyond the prognostic and predictive utility. Tumor Biol..

[20] Zhao X., Qu J., Sun Y., Wang J., Liu X., Wang F., Zhang H., Wang W., Ma X., Gao X. (2017). Prognostic significance of tumor-associated macrophages in breast cancer: A meta-analysis of the literature. Oncotarget.

[21] Wyckoff J., Wang W., Lin E.Y., Wang Y., Pixley F., Stanley E.R., Graf T., Pollard J.W., Segall J., Condeelis J. (2004). A Paracrine Loop between Tumor Cells and Macrophages Is Required for Tumor Cell Migration in Mammary Tumors. Cancer Res..

[22] Su S., Liu Q., Chen J., Chen J., Chen F., He C., Huang D., Wu W., Lin L., Huang W. (2014). A Positive Feedback Loop between Mesenchymal-like Cancer Cells and Macrophages Is Essential to Breast Cancer Metastasis. Cancer Cell.

[23] Ruffell B., Chang-Strachan D., Chan V., Rosenbusch A., Ho C.M., Pryer N., Daniel D., Hwang E.S., Rugo H.S., Coussens L.M. (2014). Macrophage IL-10 Blocks CD8+ T Cell-Dependent Responses to Chemotherapy by Suppressing IL-12 Expression in Intratumoral Dendritic Cells. Cancer Cell.

[24] Marigo I., Trovato R., Hofer F., Ingangi V., DeSantis G., Leone K., De Sanctis F., Ugel S., Cane S., Simonelli A. (2020). The Disabled homolog 2 controls pro-metastatic activity of tumor-associated macrophages. Cancer Discov..

[25] Dieci M.V., Radosevic-Robin N., Fineberg S., Eynden G.V.D., Ternes N., Penault-Llorca F., Pruneri G., D’Alfonso T.M., DeMaria S., Castaneda C. (2018). Update on tumor-infiltrating lymphocytes (TILs) in breast cancer, including recommendations to assess TILs in residual disease after neoadjuvant therapy and in carcinoma in situ: A report of the International Immuno-Oncology Biomarker Working Group on Breast Cancer. Semin. Cancer Biol..

[26] Folgueira M.A.A.K., Maistro S., Katayama M.L.H., Roela R.A., Mundim F.G.L., Nanogaki S., De Bock G.H., Brentani M.M. (2013). Markers of breast cancer stromal fibroblasts in the primary tumour site associated with lymph node metastasis: A systematic review including our case series. Biosci. Rep..

[27] Eiro N., Gonzalez L.O., Fraile M., Cid S., Schneider J., Vizoso F.J. (2019). Breast Cancer Tumor Stroma: Cellular Components, Phenotypic Heterogeneity, Intercellular Communication, Prognostic Implications and Therapeutic Opportunities. Cancers.

[28] Kos Z., Roblin E., Kim R.S., Michiels S., Gallas B.D., Chen W., Van De Vijver K.K., Goel S., Adams S., The International Immuno-Oncology Biomarker Working Group (2020). Pitfalls in assessing stromal tumor infiltrating lymphocytes (sTILs) in breast cancer. NPJ Breast Cancer.

[29] Bohling S.D., Allison K.H. (2008). Immunosuppressive regulatory T cells are associated with aggressive breast cancer phenotypes: A potential therapeutic target. Mod. Pathol..

[30] Salgado R., Denkert C., DeMaria S., Sirtaine N., Klauschen F., Pruneri G., Wienert S., Eynden G.V.D., Baehner F.L., Penault-Llorca F. (2015). The evaluation of tumor-infiltrating lymphocytes (TILs) in breast cancer: Recommendations by an International TILs Working Group 2014. Ann. Oncol..

[31] Loi S., Drubay D., Adams S., Pruneri G., Francisl P.A., Lacroix-Triki M., Joensuu H., Dieci M.V., Badve S., DeMaria S. (2019). Tumor-Infiltrating Lymphocytes and Prognosis: A Pooled Individual Patient Analysis of Early-Stage Triple-Negative Breast Cancers. J. Clin. Oncol..

[32] Michiels S., Jonas S.F., Bataillon G., Criscitiello C., Salgado R., Loi S., Viale G., Lee H.J., Dieci M.V., Kim S.-B. (2019). Prognostic value of tumor-infiltrating lymphocytes in patients with early-stage triple-negative breast cancers (TNBC) who did not receive adjuvant chemotherapy. Ann. Oncol..

[33] Dieci M., Conte P., Bisagni G., Brandes A., Frassoldati A., Cavanna L., Musolino A., Giotta F., Rimanti A., Garrone O. (2019). Association of tumor-infiltrating lymphocytes with distant disease-free survival in the ShortHER randomized adjuvant trial for patients with early HER2+ breast cancer. Ann. Oncol..

[34] Dieci M.V., Criscitiello C., Goubar A., Viale G., Conte P., Guarneri V., Ficarra G., Mathieu M.C., Delaloge S., Curigliano G. (2014). Prognostic value of tumor-infiltrating lymphocytes on residual disease after primary chemotherapy for triple-negative breast cancer: A retrospective multicenter study. Ann. Oncol..

[35] Denkert C., Von Minckwitz G., Darb-Esfahani S., Lederer B., I Heppner B., E Weber K., Budczies J., Huober J., Klauschen F., Furlanetto J. (2018). Tumour-infiltrating lymphocytes and prognosis in different subtypes of breast cancer: A pooled analysis of 3771 patients treated with neoadjuvant therapy. Lancet Oncol..

[36] Kim R.S., Song N., Gavin P.G., Salgado R., Bandos H., Kos Z., Floris G., Eynden G.G.G.M.V.D., Badve S., DeMaria S. (2019). Stromal Tumor-infiltrating Lymphocytes in NRG Oncology/NSABP B-31 Adjuvant Trial for Early-Stage HER2-Positive Breast Cancer. J. Natl. Cancer Inst..

[37] Cardoso F., Kyriakides. S., Ohno S., Penault-Llorca F., Poortmans P., Rubio I.T., Zackrisson S., Senkus E., ESMO Guidelines Committee (2019). Early breast cancer: ESMO Clinical Practice Guidelines for diagnosis, treatment and follow-up. Ann. Oncol..

[38] Yu T., Di G. (2017). Role of tumor microenvironment in triple-negative breast cancer and its prognostic significance. Chin. J. Cancer Res..

[39] Lee S., Cho E.Y., Park Y.H., Ahn J.S., Im Y.-H. (2012). Prognostic impact of FOXP3 expression in triple-negative breast cancer. Acta Oncol..

[40] Mahmoud S.M., Paish E.C., Powe D.G., Macmillan R.D., Grainge M.J., Lee A.H.S., Ellis I.O., Green A.R. (2011). Tumor-Infiltrating CD8+ Lymphocytes Predict Clinical Outcome in Breast Cancer. J. Clin. Oncol..

[41] Dieci M.V., Tsvetkova V., Griguolo G., Miglietta F., Tasca G., Giorgi C.A., Cumerlato E., Massa D., Mele M.L., Orvieto E. (2020). Integration of tumour infiltrating lymphocytes, programmed cell-death ligand-1, CD8 and FOXP3 in prognostic models for triple-negative breast cancer: Analysis of 244 stage I–III patients treated with standard therapy. Eur. J. Cancer.

[42] Doedens A.L., Stockmann C., Rubinstein M.P., Liao D., Zhang N., DeNardo D.G., Coussens L.M., Karin M., Goldrath A.W., Johnson R.S. (2010). Macrophage Expression of Hypoxia-Inducible Factor-1 Suppresses T-Cell Function and Promotes Tumor Progression. Cancer Res..

[43] Mao Y., Qu Q., Chen X., Huang O., Wu J., Shen K. (2016). The Prognostic Value of Tumor-Infiltrating Lymphocytes in Breast Cancer: A Systematic Review and Meta-Analysis. PLoS ONE.

[44] Miyashita M., Sasano H., Tamaki K., Hirakawa H., Takahashi Y., Nakagawa S., Watanabe G., Tada H., Suzuki A., Ohuchi N. (2015). Prognostic significance of tumor-infiltrating CD8+ and FOXP3+ lymphocytes in residual tumors and alterations in these parameters after neoadjuvant chemotherapy in triple-negative breast cancer: A retrospective multicenter study. Breast Cancer Res..

[45] Bottai G., Raschioni C., Losurdo A., Di Tommaso L., Tinterri C., Torrisi R., Reis-Filho J.S., Roncalli M., Sotiriou C., Santoro A. (2016). An immune stratification reveals a subset of PD-1/LAG-3 double-positive triple-negative breast cancers. Breast Cancer Res..

[46] Yeong J., Thike A.A., Lim J.C.T., Lee B., Li H., Wong S.-C., Hue S.S.S., Tan P.H., Iqbal J. (2017). Higher densities of Foxp3+ regulatory T cells are associated with better prognosis in triple-negative breast cancer. Breast Cancer Res. Treat..

[47] West N.R., E Kost S., Martin S.D., Milne K., DeLeeuw R.J., Nelson B.H., Watson P.H. (2012). Tumour-infiltrating FOXP3+ lymphocytes are associated with cytotoxic immune responses and good clinical outcome in oestrogen receptor-negative breast cancer. Br. J. Cancer.

[48] Liu S., Foulkes W.D., Leung S., Gao D., Lau S., Kos Z., O Nielsen T. (2014). Prognostic significance of FOXP3+ tumor-infiltrating lymphocytes in breast cancer depends on estrogen receptor and human epidermal growth factor receptor-2 expression status and concurrent cytotoxic T-cell infiltration. Breast Cancer Res..

[49] Schmidt M., Weyer-Elberich V., Hengstler J.G., Heimes A.-S., Almstedt K., Gerhold-Ay A., Lebrecht A., Battista M.J., Hasenburg A., Sahin U. (2018). Prognostic impact of CD4-positive T cell subsets in early breast cancer: A study based on the FinHer trial patient population. Breast Cancer Res..

[50] Schalper K.A., Velcheti V., Carvajal D., Wimberly H., Brown J., Pusztai L., Rimm D. (2014). In Situ Tumor PD-L1 mRNA Expression Is Associated with Increased TILs and Better Outcome in Breast Carcinomas. Clin. Cancer Res..

[51] Kythreotou A., Siddique A., Mauri F.A., Bower M., Pinato D.J. (2018). PD-L1. J. Clin. Pathol..

[52] Leite M.S.M., Van De Vijver K.K., Michaut M., Van Der Linden R., Hooijer G.K., Horlings H.M., Severson T.M., Mulligan A.M., Weerasooriya N., Sanders J. (2018). Assessment of PD-L1 expression across breast cancer molecular subtypes, in relation to mutation rate, BRCA1-like status, tumor-infiltrating immune cells and survival. OncoImmunology.

[53] Miglietta F., Griguolo G., Guarneri V., Dieci M.V. (2019). Programmed Cell Death Ligand 1 in Breast Cancer: Technical Aspects, Prognostic Implications, and Predictive Value. Oncology.

[54] Schmid P., Adams S., Rugo H.S., Schneeweiss A., Barrios C.H., Iwata H., Diéras V., Hegg R., Wright G.S., Seock-Ah IMpassion130 Trial Investigators (2018). Atezolizumab and Nab-Paclitaxel in Advanced Triple-Negative Breast Cancer. New Engl. J. Med..

[55] Muenst S., Schaerli A.R., Gao F., Däster S., Trella E., Droeser R.A., Muraro M.G., Zajac P., Zanetti R., Gillanders W.E. (2014). Expression of programmed death ligand 1 (PD-L1) is associated with poor prognosis in human breast cancer. Breast Cancer Res. Treat..

[56] Zhang M., Sun H., Zhao S., Wang Y., Pu H., Zhang Q. (2017). Expression of PD-L1 and prognosis in breast cancer: A meta-analysis. Oncotarget.

[57] Sa-Nguanraksa D., O-Charoenrat P. (2012). The Role of Vascular Endothelial Growth Factor A Polymorphisms in Breast Cancer. Int. J. Mol. Sci..

[58] Ghiabi P., Jiang J., Pasquier J., Maleki M., Abu-Kaoud N., Rafii S., Rafii A. (2014). Endothelial Cells Provide a Notch-Dependent Pro-Tumoral Niche for Enhancing Breast Cancer Survival, Stemness and Pro-Metastatic Properties. PLoS ONE.

[59] Ghajar C.M., Peinado H., Mori H., Matei I.R., Evason K.J., Brazier H., De Almeida D.L., Koller A., Hajjar K.A., Stainier D.Y.R. (2013). The perivascular niche regulates breast tumour dormancy. Nat. Cell Biol..

[60] Hill B.S., Sarnella A., D’Avino G., Zannetti A. (2020). Recruitment of stromal cells into tumour microenvironment promote the metastatic spread of breast cancer. Semin. Cancer Biol..

[61] Melzer C., Von Der Ohe J., Hass R. (2018). Enhanced metastatic capacity of breast cancer cells after interaction and hybrid formation with mesenchymal stroma/stem cells (MSC). Cell Commun. Signal..

[62] Dwyer R., Potter-Beirne S., Harrington K., Lowery A., Hennessy E., Murphy J., Barry F., O’Brien T., Kerin M. (2007). Monocyte Chemotactic Protein-1 Secreted by Primary Breast Tumors Stimulates Migration of Mesenchymal Stem Cells. Clin. Cancer Res..

[63] Jia Y., Chen Y., Wang Q., Jayasinghe U., Luo X., Wei Q., Wang J., Xiong H., Chen C., Xu B. (2017). Exosome: Emerging biomarker in breast cancer. Oncotarget.

[64] Wang S., Su X., Xu M., Xiao X., Li X., Li H., Keating A., Zhao R.C. (2019). Exosomes secreted by mesenchymal stromal/stem cell-derived adipocytes promote breast cancer cell growth via activation of Hippo signaling pathway. Stem Cell Res. Ther..

[65] Biswas S., Mandal G., Chowdhury S.R., Purohit S., Payne K.K., Anadon C., Gupta A., Swanson P., Yu X., Conejo-Garcia J.R. (2019). Exosomes Produced by Mesenchymal Stem Cells Drive Differentiation of Myeloid Cells into Immunosuppressive M2-Polarized Macrophages in Breast Cancer. J. Immunol..

[66] Rőszer T. (2015). Understanding the Mysterious M2 Macrophage through Activation Markers and Effector Mechanisms. Mediat. Inflamm..

[67] Bartosh T.J., Ullah M., Zeitouni S., Beaver J., Prockop D.J. (2016). Cancer cells enter dormancy after cannibalizing mesenchymal stem/stromal cells (MSCs). Proc. Natl. Acad. Sci. USA.

[68] Ghajar C.M. (2015). Metastasis prevention by targeting the dormant niche. Nat. Rev. Cancer.

[69] He M.-F., Wang S., Wang Y., Wang X.-N. (2013). Modeling cell-in-cell structure into its biological significance. Cell Death Dis..

[70] Chen Y.-C., Gonzalez M.E., Burman B., Zhao X., Anwar T., Tran M., Medhora N., Hiziroglu A.B., Lee W., Cheng Y.-H. (2019). Mesenchymal Stem/Stromal Cell Engulfment Reveals Metastatic Advantage in Breast Cancer. Cell Rep..

[71] Pickup M.W., Mouw J.K., Weaver V.M. (2014). The extracellular matrix modulates the hallmarks of cancer. EMBO Rep..

[72] Gole L., Yeong J., Lim J.C.T., Ong K.H., Han H., Thike A.A., Poh Y.C., Yee S., Iqbal J., Hong W. (2020). Quantitative stain-free imaging and digital profiling of collagen structure reveal diverse survival of triple negative breast cancer patients. Breast Cancer Res..

[73] Oskarsson T. (2013). Extracellular matrix components in breast cancer progression and metastasis. Breast.

[74] Duffy M.J., Maguire T.M., Hill A., McDermott E., O’Higgins N. (2000). Metalloproteinases: Role in breast carcinogenesis, invasion and metastasis. Breast Cancer Res..

[75] Radisky E.S., Radisky D.C. (2015). Matrix metalloproteinases as breast cancer drivers and therapeutic targets. Front. Biosci..

[76] Provenzano P.P., Eliceiri K.W., Campbell J.M., Inman D.R., White J.G., Keely P.J. (2006). Collagen reorganization at the tumor-stromal interface facilitates local invasion. BMC Med..

[77] Conklin M.W., Eickhoff J.C., Riching K.M., Pehlke C.A., Eliceiri K.W., Provenzano P.P., Friedl A., Keely P.J. (2011). Aligned Collagen Is a Prognostic Signature for Survival in Human Breast Carcinoma. Am. J. Pathol..

[78] Guiro K., Arinzeh T.L. (2015). Bioengineering Models for Breast Cancer Research. Breast Cancer: Basic Clin. Res..

[79] Nath S., Devi G.R. (2016). Three-dimensional culture systems in cancer research: Focus on tumor spheroid model. Pharmacol. Ther..

[80] Drost J., Clevers H. (2018). Organoids in cancer research. Nat. Rev. Cancer.

[81] Weiswald L.-B., Bellet D., Dangles-Marie V. (2015). Spherical Cancer Models in Tumor Biology. Neoplasia.

[82] Liu Z., Vunjak-Novakovic G. (2016). Modeling tumor microenvironments using custom-designed biomaterial scaffolds. Curr. Opin. Chem. Eng..

[83] Cavallaro S. (2013). CXCR4/CXCL12 in non-small-cell lung cancer metastasis to the brain. Int. J. Mol. Sci..

[84] Yao Q., Xu C., Zhao H., Chen H. (2015). CXCR4 in breast cancer: Oncogenic role and therapeutic targeting. Drug Des. Dev. Ther..

[85] Dewan M., Ahmed S., Iwasaki Y., Ohba K., Toi M., Yamamoto N. (2006). Stromal cell-derived factor-1 and CXCR4 receptor interaction in tumor growth and metastasis of breast cancer. Biomed. Pharmacother..

[86] Angst B.D., Marcozzi C., I Magee A. (2001). The cadherin superfamily: Diversity in form and function. J. Cell Sci..

[87] Andrews J.L., Kim A.C., Hens J. (2012). The role and function of cadherins in the mammary gland. Breast Cancer Res..

[88] Shah S.H., Miller P., Garcia-Contreras M., Ao Z., Machlin L., Issa E., El-Ashry D. (2015). Hierarchical paracrine interaction of breast cancer associated fibroblasts with cancer cells via hMAPK-microRNAs to drive ER-negative breast cancer phenotype. Cancer Biol. Ther..

[89] Jaalouk D.E., Lammerding J. (2009). Mechanotransduction gone awry. Nat. Rev. Mol. Cell Biol..

[90] Chin L., Xia Y., E Discher D., A Janmey P. (2016). Mechanotransduction in cancer. Curr. Opin. Chem. Eng..

[91] Huang S., Ingber D.E. (2005). Cell tension, matrix mechanics, and cancer development. Cancer Cell.

[92] Wolf K., Wu Y.I., Liu Y., Geiger J., Tam E., Overall C.M., Stack M.S., Friedl P. (2007). Multi-step pericellular proteolysis controls the transition from individual to collective cancer cell invasion. Nat. Cell Biol..

[93] Suresh S. (2007). Biomechanics and biophysics of cancer cells. Acta Biomater..

[94] Paszek M.J., Zahir N., Johnson K.R., Lakins J.N., Rozenberg G.I., Gefen A., Reinhart-King C.A., Margulies S.S., Dembo M., Boettiger D. (2005). Tensional homeostasis and the malignant phenotype. Cancer Cell.

[95] Gaggioli C., Hooper S., Hidalgo-Carcedo C., Grosse R., Marshall J.F., Harrington K., Sahai E. (2007). Fibroblast-led collective invasion of carcinoma cells with differing roles for RhoGTPases in leading and following cells. Nat. Cell Biol..

[96] Hebner C., Weaver V.M., Debnath J. (2008). Modeling Morphogenesis and Oncogenesis in Three-Dimensional Breast Epithelial Cultures. Annu. Rev. Pathol. Mech. Dis..

[97] Panciera T., Azzolin L., Cordenonsi M., Piccolo S. (2017). Mechanobiology of YAP and TAZ in physiology and disease. Nat. Rev. Mol. Cell Biol..

[98] Calses P.C., Crawford J.J., Lill J.R., Dey A. (2019). Hippo Pathway in Cancer: Aberrant Regulation and Therapeutic Opportunities. Trends Cancer.

[99] Ma S., Meng Z., Chen R., Guan K.-L. (2019). The Hippo Pathway: Biology and Pathophysiology. Annu. Rev. Biochem..

[100] Harvey K.F., Tapon N. (2007). The Salvador–Warts–Hippo pathway—An emerging tumour-suppressor network. Nat. Rev. Cancer.

[101] Zhao B., Li L., Tumaneng K., Wang C.Y., Guan K.L. (2010). A coordinated phosphorylation by Lats and CK1 regulates YAP stability through SCF(beta-TRCP). Genes Dev..

[102] Totaro A., Panciera T., Piccolo S. (2018). YAP/TAZ upstream signals and downstream responses. Nat. Cell Biol..

[103] Zanconato F., Cordenonsi M., Piccolo S. (2019). YAP and TAZ: A signalling hub of the tumour microenvironment. Nat. Rev. Cancer.

[104] Zanconato F., Cordenonsi M., Piccolo S. (2016). YAP/TAZ at the Roots of Cancer. Cancer Cell.

[105] Wu L., Yang X. (2018). Targeting the Hippo Pathway for Breast Cancer Therapy. Cancers.

[106] Lamar J.M., Stern P., Liu H., Schindler J.W., Jiang Z.-G., Hynes R.O. (2012). The Hippo pathway target, YAP, promotes metastasis through its TEAD-interaction domain. Proc. Natl. Acad. Sci. USA.

[107] Overholtzer M., Zhang J., Smolen G.A., Muir B., Li W., Sgroi D.C., Deng C.-X., Brugge J.S., Haber D.A. (2006). Transforming properties of YAP, a candidate oncogene on the chromosome 11q22 amplicon. Proc. Natl. Acad. Sci. USA.

[108] Lee J.Y., Chang J.K., Dominguez A.A., Lee H.-P., Nam S., Chang J., Varma S., Qi L.S., West R.B., Chaudhuri O. (2019). YAP-independent mechanotransduction drives breast cancer progression. Nat. Commun..

[109] Chen Q., Zhang N., Gray R.S., Li H., Ewald A.J., Zahnow C.A., Pan D. (2014). A temporal requirement for Hippo signaling in mammary gland differentiation, growth, and tumorigenesis. Genes Dev..

[110] Bartucci M., Dattilo R., Moriconi C., Pagliuca A., Mottolese M., Federici G., Di Benedetto A., Todaro M., Stassi G., Sperati F. (2014). TAZ is required for metastatic activity and chemoresistance of breast cancer stem cells. Oncogene.

[111] Díaz-Martín J., López-García M.Á., Romero-Pérez L., Atienza-Amores M.R., Pecero M.L., Castilla M.Á., Biscuola M., Santón A., Palacios J., Atienza M.R. (2015). Nuclear TAZ expression associates with the triple-negative phenotype in breast cancer. Endocr. Relat. Cancer.

[112] Li Y.-W., Shen H., Frangou C., Yang N., Guo J., Xu B., Bshara W., Shepherd L., Zhu Q., Wang J. (2015). Characterization of TAZ domains important for the induction of breast cancer stem cell properties and tumorigenesis. Cell Cycle.

[113] Skibinski A., Breindel J.L., Prat A., Galván P., Smith E., Rolfs A., Gupta P.B., LaBaer J., Kuperwasser C. (2014). The Hippo Transducer TAZ Interacts with the SWI/SNF Complex to Regulate Breast Epithelial Lineage Commitment. Cell Rep..

[114] Vici P., Mottolese M., Pizzuti L., Barba M., Sperati F., Terrenato I., Di Benedetto A., Natoli C., Gamucci T., Angelucci D. (2014). The Hippo transducer TAZ as a biomarker of pathological complete response in HER2-positive breast cancer patients treated with trastuzumab-based neoadjuvant therapy. Oncotarget.

[115] Yoon A.-R., Stasinopoulos I., Kim J.H., Yong H.M., Kilic O., Wirtz D., Bhujwalla Z.M., An S.S. (2015). COX-2 dependent regulation of mechanotransduction in human breast cancer cells. Cancer Biol. Ther..

[116] Ao Z., Shah S.H., Machlin L.M., Parajuli R., Miller P.C., Rawal S., Williams A.J., Cote R.J., Lippman M.E., Datar R.H. (2015). Identification of Cancer-Associated Fibroblasts in Circulating Blood from Patients with Metastatic Breast Cancer. Cancer Res..

[117] Eynden G.G.V.D., Majeed A.W., Illemann M., Vermeulen P., Bird N.C., Høyer-Hansen G., Eefsen R.L., Reynolds A.R., Brodt P. (2013). The Multifaceted Role of the Microenvironment in Liver Metastasis: Biology and Clinical Implications. Cancer Res..

[118] Ma R., Feng Y.-L., Lin S., Chen J., Lin H., Liang X., Zheng H., Cai X. (2015). Mechanisms involved in breast cancer liver metastasis. J. Transl. Med..

[119] Saunus J.M., Reed A.E.M., Lim Z.L., Lakhani S.R. (2017). Breast Cancer Brain Metastases: Clonal Evolution in Clinical Context. Int. J. Mol. Sci..

[120] Neman J., Choy C., Kowolik C.M., Anderson A., Duenas V.J., Waliany S., Chen B.T., Chen M.Y., Jandial R. (2013). Co-evolution of breast-to-brain metastasis and neural progenitor cells. Clin. Exp. Metastasis.

[121] Kang Y. (2016). Dissecting Tumor-Stromal Interactions in Breast Cancer Bone Metastasis. Endocrinol. Metab..

[122] Kolb A.D., Bussard K.M. (2019). The Bone Extracellular Matrix as an Ideal Milieu for Cancer Cell Metastases. Cancers.

[123] Network T.C.G.A. (2012). Comprehensive molecular portraits of human breast tumours. Nat. Cell Biol..

[124] Dent R., Trudeau M., Pritchard K.I., Hanna W.M., Kahn H.K., Sawka C.A., Lickley L.A., Rawlinson E., Sun P., Narod S.A. (2007). Triple-Negative Breast Cancer: Clinical Features and Patterns of Recurrence. Clin. Cancer Res..

[125] Perou C.M., Sørlie T., Eisen M.B., Van De Rijn M., Jeffrey S.S., A Rees C., Pollack J.R., Ross D.T., Johnsen H., Akslen L.A. (2000). Molecular portraits of human breast tumours. Nat. Cell Biol..

[126] Kreike B., Van Kouwenhove M., Horlings H., Weigelt B., Peterse H., Bartelink H., Van De Vijver M.J. (2007). Gene expression profiling and histopathological characterization of triple-negative/basal-like breast carcinomas. Breast Cancer Res..

[127] Alluri P., Newman L.A. (2014). Basal-Like and Triple-Negative Breast Cancers. Surg. Oncol. Clin. North Am..

[128] Weigelt B., Baehner F.L., Reis-Filho J.S. (2010). The contribution of gene expression profiling to breast cancer classification, prognostication and prediction: A retrospective of the last decade. J. Pathol..

[129] Dieci M.V., Mathieu M.C., Guarneri V., Conte P., Delaloge S., Andre F., Goubar A. (2015). Prognostic and predictive value of tumor-infiltrating lymphocytes in two phase III randomized adjuvant breast cancer trials. Ann. Oncol..

[130] Stanton S.E., Disis M.L. (2016). Clinical significance of tumor-infiltrating lymphocytes in breast cancer. J. Immunother. Cancer.

[131] Rakha E.A., Ellis I.O. (2009). Triple-negative/basal-like breast cancer: Review. Pathology.

[132] Stanton S.E., Adams S., Disis M.L. (2016). Variation in the Incidence and Magnitude of Tumor-Infiltrating Lymphocytes in Breast Cancer Subtypes. JAMA Oncol..

[133] Liu F., Lang R., Zhao J., Zhang X., Pringle G.A., Fan Y., Yin D., Gu F., Yao Z., Fu F. (2011). CD8^+^ cytotoxic T cell and FOXP3^+^ regulatory T cell infiltration in relation to breast cancer survival and molecular subtypes. Breast Cancer Res. Treat..

[134] Miyan M., Schmidt-Mende J., Kiessling R., Poschke I., De Boniface J. (2016). Differential tumor infiltration by T-cells characterizes intrinsic molecular subtypes in breast cancer. J. Transl. Med..

[135] Glajcar A., Szpor J., Pacek A., Tyrak K.E., Chan F., Streb J., Hodorowicz-Zaniewska D., Okoń K. (2017). The relationship between breast cancer molecular subtypes and mast cell populations in tumor microenvironment. Virchows Arch..

[136] Medrek C., Pontén F., Jirström K., Leandersson K. (2012). The presence of tumor associated macrophages in tumor stroma as a prognostic marker for breast cancer patients. BMC Cancer.

[137] Sousa S., Brion R., Lintunen M., Kronqvist P., Sandholm J., Mönkkönen J., Kellokumpu-Lehtinen P.-L., Lauttia S., Tynninen O., Joensuu H. (2015). Human breast cancer cells educate macrophages toward the M2 activation status. Breast Cancer Res..

[138] Hollmén M., Karaman S., Schwager S., Lisibach A., Christiansen A.J., Maksimow M., Varga Z., Jalkanen S., Detmar M. (2015). G-CSF regulates macrophage phenotype and associates with poor overall survival in human triple-negative breast cancer. OncoImmunology.

[139] Levano K.S., Jung E.H., Kenny P.A. (2011). Breast cancer subtypes express distinct receptor repertoires for tumor-associated macrophage derived cytokines. Biochem. Biophys. Res. Commun..

[140] Niemiec J.A., Adamczyk A., Ambicka A., Mucha-Małecka A., Wysocki W.M., Ryś J. (2014). Triple-negative, Basal Marker-expressing, and High-grade Breast Carcinomas are Characterized by High Lymphatic Vessel Density and the Expression of Podoplanin in Stromal Fibroblasts. Appl. Immunohistochem. Mol. Morphol..

[141] Linderholm B.K., Hellborg H., Johansson U., Elmberger G., Skoog L., Lehtiö J., Lewensohn R. (2009). Significantly higher levels of vascular endothelial growth factor (VEGF) and shorter survival times for patients with primary operable triple-negative breast cancer. Ann. Oncol..

[142] Sa-Nguanraksa D., Chuangsuwanich T., Pongpruttipan T., O-Charoenrat P. (2015). High vascular endothelial growth factor gene expression predicts poor outcome in patients with non-luminal A breast cancer. Mol. Clin. Oncol..

[143] Schneider B.P., Gray R.J., Radovich M., Shen F., Vance G., Li L., Jiang G., Miller K.D., Gralow J.R., Dickler M.N. (2013). Prognostic and predictive value of tumor vascular endothelial growth factor gene amplification in metastatic breast cancer treated with paclitaxel with and without bevacizumab; results from ECOG 2100 trial. Clin. Cancer Res..

[144] Sihto H., Lundin J., Lundin M., Lehtimäki T., Ristimäki A., Holli K., Sailas L., Kataja V., Turpeenniemi-Hujanen T., Isola J. (2011). Breast cancer biological subtypes and protein expression predict for the preferential distant metastasis sites: A nationwide cohort study. Breast Cancer Res..

[145] Smid M., Wang Y., Zhang Y., Sieuwerts A.M., Yu J., Klijn J.G.M., Foekens J.A., Martens J.W.M. (2008). Subtypes of Breast Cancer Show Preferential Site of Relapse. Cancer Res..

[146] Kennecke H., Yerushalmi R., Woods R., Cheang M.C.U., Voduc D., Speers C.H., Nielsen T.O., Gelmon K. (2010). Metastatic Behavior of Breast Cancer Subtypes. J. Clin. Oncol..

[147] Soni D.A., Ren Z., Hameed O., Chanda D., Morgan C.J., Siegal G.P., Wei S. (2015). Breast Cancer Subtypes Predispose the Site of Distant Metastases. Am. J. Clin. Pathol..

[148] Emens L.A., Nanda R. (2018). Breast Cancer Immunotherapy. Cancer Immunother. Princ. Pract..

[149] DeNardo D.G., Brennan D.J., Rexhepaj E., Ruffell B., Shiao S.L., Madden S.F., Gallagher W.M., Wadhwani N., Keil S.D., Junaid S.A. (2011). Leukocyte Complexity Predicts Breast Cancer Survival and Functionally Regulates Response to Chemotherapy. Cancer Discov..

[150] Zhong Z., Carroll K.D., Policarpio D., Osborn C., Gregory M., Bassi R., Jimenez X., Prewett M., Liebisch G., Persaud K. (2010). Anti-transforming growth factor beta receptor II antibody has therapeutic efficacy against primary tumor growth and metastasis through multieffects on cancer, stroma, and immune cells. Clin. Cancer Res..

[151] Chen X., Yang Y., Zhou Q., Weiss J.M., Howard O.Z., McPherson J.M., Wakefield L.M., Oppenheim J.J. (2014). Effective Chemoimmunotherapy with Anti-TGFβ Antibody and Cyclophosphamide in a Mouse Model of Breast Cancer. PLoS ONE.

[152] Wang Y.-J., Fletcher R., Yu J., Zhang L. (2018). Immunogenic effects of chemotherapy-induced tumor cell death. Genes Dis..

[153] DeMaria S., Ng B., Devitt M.L., Babb J.S., Kawashima N., Liebes L., Formenti S.C. (2004). Ionizing radiation inhibition of distant untreated tumors (abscopal effect) is immune mediated. Int. J. Radiat. Oncol..

[154] Voorwerk L., Slagter M., Horlings H.M., Sikorska K., Van De Vijver K.K., De Maaker M., Nederlof I., Kluin R.J.C., Warren S., Ong S. (2019). Immune induction strategies in metastatic triple-negative breast cancer to enhance the sensitivity to PD-1 blockade: The TONIC trial. Nat. Med..

[155] Svensson S., Abrahamsson A., Rodriguez G.V., Olsson A.-K., Jensen L., Cao Y., Dabrosin C. (2015). CCL2 and CCL5 Are Novel Therapeutic Targets for Estrogen-Dependent Breast Cancer. Clin. Cancer Res..

[156] Generali D., Bates G., Berruti A., Brizzi M.P., Campo L., Bonardi S., Bersiga A., Allevi G., Milani M., Aguggini S. (2009). Immunomodulation of FOXP3+ Regulatory T Cells by the Aromatase Inhibitor Letrozole in Breast Cancer Patients. Clin. Cancer Res..

[157] Chan M.S.M., Wang L., A Felizola S.J., Ueno T., Toi M., Loo W., Chow L.W.C., Suzuki T., Sasano H. (2012). Changes of tumor infiltrating lymphocyte subtypes before and after neoadjuvant endocrine therapy in estrogen receptor-positive breast cancer patients–an immunohistochemical study of cd8+ and foxp3+ using double immunostaining with correlation to the pathobiological response of the patients. Int. J. Biol. Markers.

[158] Nalbandian G., Paharkova-Vatchkova V., Mao A., Nale S., Kovats S. (2005). The Selective Estrogen Receptor Modulators, Tamoxifen and Raloxifene, Impair Dendritic Cell Differentiation and Activation. J. Immunol..

[159] Komi J., Lassila O. (2000). Nonsteroidal anti-estrogens inhibit the functional differentiation of human monocyte-derived dendritic cells. Blood.

[160] Behjati S., Frank M.H. (2009). The Effects of Tamoxifen on Immunity. Curr. Med. Chem..

[161] EBCTCG (2015). Adjuvant bisphosphonate treatment in early breast cancer: Meta-analyses of individual patient data from randomised trials. Lancet.

[162] Wang X., Yang K.H., Wanyan P., Tian J.H. (2014). Comparison of the efficacy and safety of denosumab versus bisphosphonates in breast cancer and bone metastases treatment: A meta-analysis of randomized controlled trials. Oncol. Lett..

